# IgG subclasses and their significance in pemphigus disease progression monitoring

**DOI:** 10.3389/fimmu.2026.1895265

**Published:** 2026-07-29

**Authors:** Yi Wang, Shan Yue, Yanqing He, Meng Wang, Xiaoting Zhao, Xu Sang, Gongqi Yu, Chuan Wang, Hong Liu, Furen Zhang

**Affiliations:** 1 Department of Clinical Laboratory, Dermatology Hospital of Shandong First Medical University, Jinan, Shandong, China; 2 Shandong Provincial Institute of Dermatology and Venereology, Shandong Academy of Medical Sciences, Jinan, Shandong, China

**Keywords:** desmoglein antibodies, disease activity, IgG subclasses, marker, pemphigus

## Abstract

**Background:**

Immunoglobulin G (IgG) subclasses have been documented to have a close association with pemphigus. Nevertheless, the relationship among IgG subclasses, anti-desmoglein (anti-Dsg) autoantibodies, and disease activity remains ambiguous, and their potential utility in monitoring disease progression has yet to be fully clarified.

**Objective:**

This study aims to unravel the associations among IgG subclasses, autoantibodies, and disease activity, thereby offering specific serological evidence to facilitate accurate disease assessment.

**Methods:**

Confirmed pemphigus patients (n=183) and healthy controls (n=38) were enrolled. Chemiluminescent immunoassay (CLIA) was used to measure serum levels of IgG1, IgG2, IgG3 and IgG4 in all participants. For pemphigus patients, the levels of anti-Dsg1 and Dsg3 autoantibodies were detected using enzyme-linked immunosorbent assay (ELISA). The correlations of IgG subclasses with anti-Dsg1 and Dsg3 autoantibodies, and pemphigus disease area index (PDAI) were evaluated in different disease stages.

**Results:**

Overall, pemphigus patients showed significantly lower levels of IgG3 and IgG4 (*P =* 0.022 and *P =* 0.040, respectively) compared with healthy controls. IgG subclasses exhibited a notable correlation with autoantibody levels, particularly during treatment. In patients with single positivity for anti-Dsg1, all IgG subclasses were lower in the remission group than in the healthy controls and active group. In contrast, among patients with double positivity for anti-Dsg1/3, IgG3 levels were significantly lower in the remission group. In patients with single positivity for anti-Dsg3, IgG2 levels differed markedly between the active group and the other groups. Receiver operating characteristic (ROC) curve identified an IgG2 cutoff of 5224 (AUC = 0.7277) for anti-Dsg3 single-positive patients and an IgG3 cutoff of 189 (AUC = 0.7596) for anti-Dsg1/Dsg3 double-positive patients to distinguish active and remission pemphigus.

**Conclusion:**

IgG2 and IgG3 may serve as valuable adjunctive markers to existing assays for Dsg antibodies. Detection of IgG2 and IgG3 could serve as effective serological tools for judging disease status and facilitating individualized clinical assessment.

## 1 Introduction

Pemphigus is a chronic autoimmune bullous dermatosis mediated by autoantibodies, which involves the skin and mucous membranes and exerts severe impacts on patients’ physical and mental health. Clinically, it is mainly classified into two subtypes: pemphigus vulgaris (PV) and pemphigus foliaceus (PF) (1, 2). The primary pathogenic antibodies of pemphigus are IgG antibodies that target desmoglein 1 (Dsg1) and desmoglein 3 (Dsg3). Affected individuals are mostly aged 36-72, with a slight female predominance in most regions. The global annual incidence is 1–7 cases per million. In the US, the mortality rate is 0.021 per 100,000 inhabitants, and patients have a 2.6-3.3-fold higher death risk than age-matched peers (3–8).

Alterations in serum immunoglobulins and complement components have been reported in pemphigus patients, suggesting systemic humoral involvement beyond skin lesions (9). Because serum immunoglobulin and complement levels correlate with disease features in some cohorts, monitoring these markers may aid clinical assessment (9). IgG antibodies are categorized into four distinct subclasses: IgG1, IgG2, IgG3, and IgG4. Research has revealed that various autoimmune diseases exhibit specific correlations with particular IgG subclasses. For instance, during the active phase of systemic lupus erythematosus, levels of IgG1 and IgG4 are notably elevated (10). Similarly, in the active stage of rheumatoid arthritis, there is an increase in IgG1 and IgG3 levels (11). Furthermore, in IgG4-related disease, a rebound in IgG4 levels post-treatment serves as an indicator of disease recurrence (12).

In pemphigus, anti-Dsg1 autoantibody titers are more reliable and informative for monitoring disease extent and activity, especially in patients with mucocutaneous involvement. By contrast, anti-Dsg3 autoantibody titers are primarily valuable for diagnostic purposes but do not reliably reflect disease activity (13–15). Consistent with this, *Pascal Joly* et al. reported that the utility of anti-Dsg3 antibody levels in monitoring disease activity in pemphigus remains suboptimal and imperfect. Notably, in patients with PV, anti-Dsg3 antibody results should never be used in isolation to guide therapeutic decisions, particularly in the absence of corresponding clinical manifestations (16). Furthermore, IgG is the primary antibody subclass that mediates pemphigus pathogenesis; however, its dynamic changes during disease progression and associations with clinical phenotypes have not been fully elucidated. Accordingly, we systematically analyzed the dynamic profiles of individual IgG subclasses across different disease stages (baseline, active stage, and remission) and among distinct antibody-positive subgroups (anti-Dsg1 single-positive, anti-Dsg3 single-positive, and anti-Dsg1/Dsg3 double-positive), and explored their correlations with disease activity. This study aimed to clarify the associations between IgG subclasses and pemphigus progression, with the goal of providing novel serological evidence to support accurate clinical disease evaluation.

## 2 Patients and methods

### 2.1 Patients and samples

Serum samples in this study were collected from patients with pemphigus who received treatment at the Dermatology Hospital of Shandong First Medical University within the past 2 year. All patients were diagnosed with pemphigus by dermatologists based on clinical manifestations, histopathological examinations, and immunological tests, in accordance with the clinical diagnosis and treatment standards for pemphigus. Meanwhile, 38 healthy individuals were enrolled as the healthy control group.

Based on the status of disease activity, patients suffering from pemphigus were categorized into three distinct disease stages: the baseline stage, the active stage, and the remission stage. Specifically, the baseline stage was defined as the clinical and serological status within 7 days prior to the initiation of systemic treatment. The active stage was defined by the presence of new blisters or erosions, an elevated PDAI score, and/or increased anti-Dsg1/3 autoantibody titers. In contrast, remission was defined as the absence of new blisters and erosions, complete re-epithelialization of all preexisting lesions, and sustained lesion-free status for at least 4 consecutive weeks with a PDAI score of 0, even though their serum Dsg1/Dsg3 antibody titers remain positive. These definitions were consistent with the 2024 Chinese Guidelines for the Diagnosis and Management of Pemphigus (17). In total, this study incorporated 183 pemphigus patients who fulfilled the aforementioned diagnostic and staging criteria. This study was approved by the Ethics Committee of Dermatology Hospital of Shandong First Medical University.

### 2.2 Materials and methods

#### 2.2.1 Detection of IgG subclasses

Antibody titers against IgG subclasses were measured using the chemiluminescent immunoassay (CLIA) method, using the immunoglobulin G assay kits (C86086G, C86087G, C86088C, C86089G, Shenzhen YHLO Biotechnology). This method is fully automated and integrated with the iFlash system (Shenzhen YHLO Biotechnology). The specific operational procedures are detailed in the product instruction manual of the aforementioned IgG assay kit.

#### 2.2.2 Detection of anti-Dsg1 and Dsg3 antibody

Enzyme-linked immunosorbent assay (ELISA) was employed to detect the titers of anti-Dsg1 and Dsg3 antibody. The detection was carried out using anti-desmoglein 1/3 antibody IgG detection kit (EA 1495–4801 G, EA 1496–4801 G, Euroimmun, Germany). The entire detection process was integrated with a fully automatic indirect immunofluorescence/enzyme-linked immunoassay analyzer (SprinterXL, Euroimmun, Germany). The specific operational procedures are detailed in the product instruction manual of the aforementioned assay kit.

#### 2.2.3 Statistical analysis

All statistical analyses were performed using SPSS 26.0 software. Descriptive statistics were applied for baseline characterization. Continuous variables are presented as the median and interquartile range [M (Q1, Q3)], whereas categorical variables are summarized as frequencies and percentages (%). For between-group comparisons of continuous variables, the two-tailed unpaired Student’s *t-test* or the Mann-Whitney U test was used as appropriate. One-way analysis of variance (ANOVA) or the nonparametric Kruskal-Wallis H test was adopted for comparisons among three or more groups. Spearman correlation analysis was conducted to evaluate the relationships between IgG subclasses and clinical disease parameters. A two-sided P value < 0.05 was considered statistically significant. Significance levels were labeled as follows: *P < 0.05, **P < 0.01, ***P < 0.001, and *ns*, no significant difference.

## 3 Results

### 3.1 Analysis of differences in IgG subclass levels and their correlation with autoantibodies

**Figure 1:**
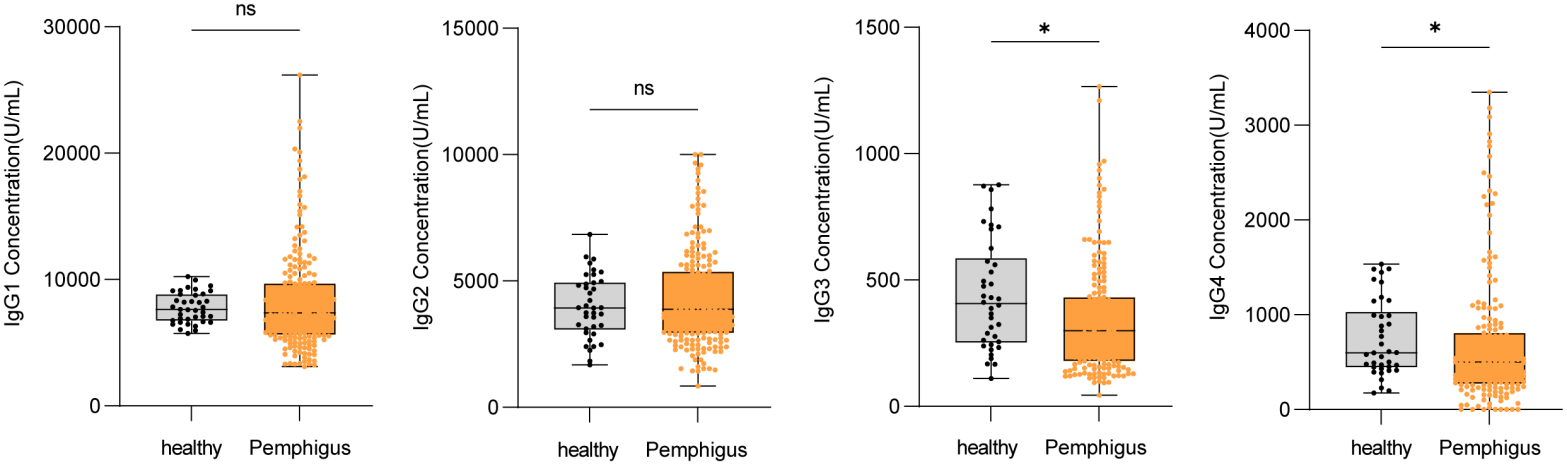
Differential analysis of IgG subclasses between pemphigus patients and healthy controls. Box plots combined with scatter plots were used to show the distribution differences in serum concentrations of IgG1, IgG2, IgG3, and IgG4 between pemphigus patients and healthy controls, respectively. ns, not significant; *P < 0.05.

183 pemphigus patients and 38 healthy controls were enrolled in this study. The median age of the patients was 58.00 years, including 89 males (48.6%) and 94 females (51.4%). Detailed baseline information covering disease stages, anti-Dsg antibody profiles and lesion distribution is summarized in **Table 1**. The serum levels of IgG subclasses were measured in all participants, and the results showed that the serum level of IgG3 in pemphigus patients was significantly lower than that in the healthy control group (*P =* 0.022), and the serum level of IgG4 in pemphigus patients was also significantly lower than that in the healthy control group (*P =* 0.040) (**Figure 1**). There were no significant differences in the levels of IgG1 (*P =* 0.531) and IgG2 (*P =* 0.600) between the two groups (**Figure 1**). These findings suggest that the abnormal immunoglobulin levels in pemphigus patients are mainly concentrated in IgG3 and IgG4.

**Table 1:** Baseline characteristics of pemphigus patients.

Baseline indicators	Statistical results
Age, median (IQR)	58.00 (48.00, 67.00)
Gender	
Male (n %)	89 (48.6%)
Female (n %)	94 (51.4%)
Disease stage n (%)	
Baseline	63 (34.4%)
Active stage	80 (43.7%)
Remission stage	40 (21.9%)
Anti-Dsg antibody profile n (%)	
anti-Dsg1 single-positive	53 (29.0%)
anti-Dsg3 single-positive	37 (20.2%)
anti-Dsg1/Dsg3 double-positive	93 (50.8%)
Lesion sites (n%)	
Oral cavity	40 (21.9%)
Head	17 (9.3%)
Limbs	6 (3.3%)
Chest and back	27 (14.8%)
Whole body	73 (39.9%)
Whole body, Oral cavity	20 (10.9%)

**Figure 2:**
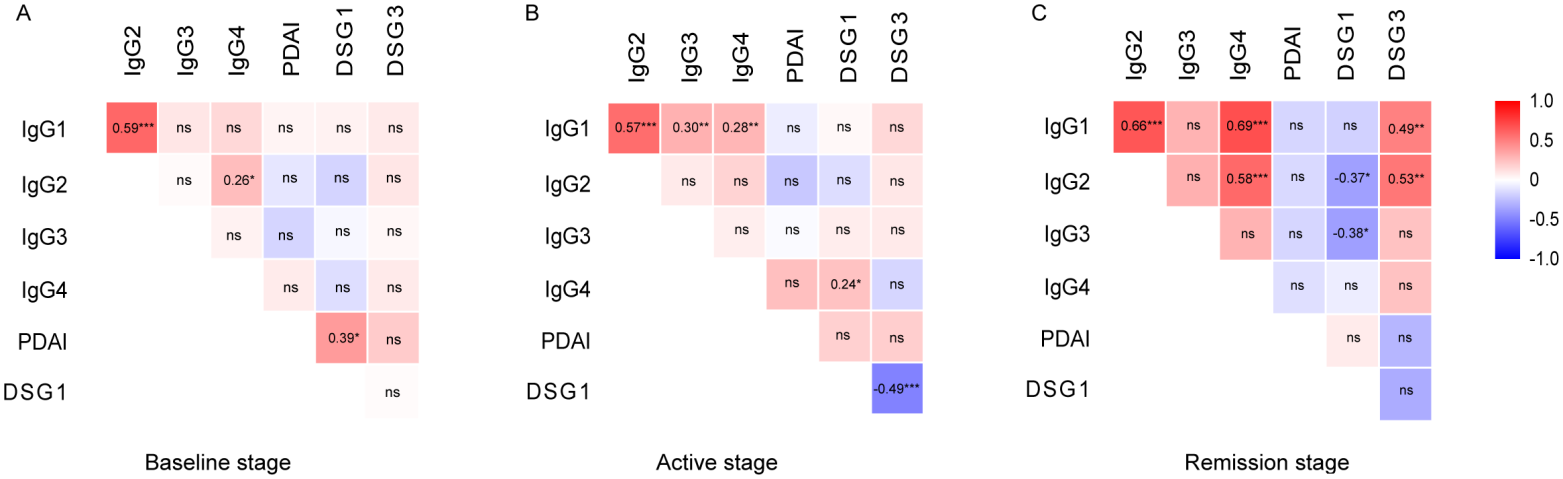
Correlation analysis among IgG subclasses, anti-Dsg antibodies and PDAI. Spearman correlation heatmaps **(A)** baseline stage, **(B)** active stage, **(C)** remission stage are shown to illustrate the correlations between serum IgG subclasses, pemphigus-specific antibodies, and PDAI in pemphigus patients at the baseline stage, active stage, and remission stage. The color intensity and hue (red for positive correlation, blue for negative correlation) in the heatmaps represent the magnitude and direction of the Spearman correlation coefficients, respectively.

To clarify the relationships between IgG subclasses and pemphigus-specific antibodies (anti-Dsg1, anti-Dsg3), as well as disease activity, correlation analyses were respectively conducted between IgG1, IgG2, IgG3, IgG4, anti-Dsg1 antibodies, anti-Dsg3 antibodies, and the Pemphigus Disease Activity Index (PDAI) in pemphigus patients at the baseline, active, and remission stages. The results revealed that there is no significant correlation between IgG subclasses and anti-Dsg1 and Dsg3 antibodies, while PDAI was positively correlated with anti-Dsg1 antibodies (*r =* 0.39, *P <* 0.05) in pemphigus patients at the baseline stage (**Figure 2A**). At the active stage, IgG4 showed a significant positive correlation with anti-Dsg1 antibodies (*r =* 0.24, *P <* 0.05). In addition, anti-Dsg1 levels presented a prominent moderate negative correlation with anti-Dsg3 titers among active patients (*r =* -0.49, *P <* 0.001) (**Figure 2B**). The most significant correlation was observed at the remission stage. Both IgG1 and IgG2 were positively correlated with anti-Dsg3 antibody (*r =* 0.49*, P <* 0.01; *r =* 0.53*, P <* 0.01), while IgG2 and IgG3 were negatively correlated with anti-Dsg1 antibody (*r =* -0.37*, P <* 0.05*; r =* -0.38, P < 0.05) (**Figure 2C**). This indicates that the close association between IgG subclasses and anti-Dsg antibodies provides another potential tool for clinical monitoring of pemphigus, particularly for the disease treatment period.

### 3.2 Differential analysis of IgG subclasses among pemphigus patients with different antibody-positive statuses

**Figure 3:**
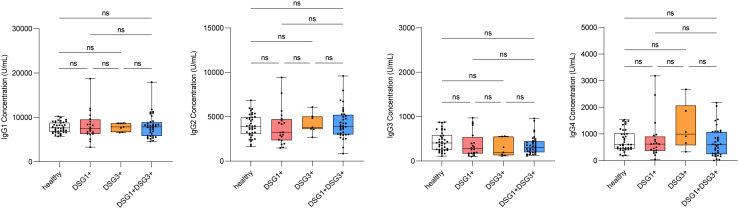
Differential analysis of IgG subclasses among pemphigus patients with different antibody-positive statuses during the baseline stage. Box plots combined with scatter plots were used to show the distribution differences of serum IgG1, IgG2, IgG3, and IgG4 among healthy controls, anti-Dsg1 single-positive, anti-Dsg3 single-positive, and anti-Dsg1/Dsg3 double-positive patients at the baseline stage. ns, not significant.

Given the significant association between IgG subclasses and anti-Dsg antibodies titers, this study performed a differential analysis of IgG1, IgG2, IgG3 and IgG4 in four groups (healthy controls, anti-Dsg1 single-positive patients, anti-Dsg3 single-positive patients, and anti-Dsg1/anti-Dsg3 double-positive patients) across three disease stages (baseline stage, active stage, and remission stage). Results at the baseline stage revealed no significant differences in serum IgG subclasses between different Dsg antibody-positive groups and the healthy control group (*P >* 0.05) (**Figure 3**).

**Figure 4:**
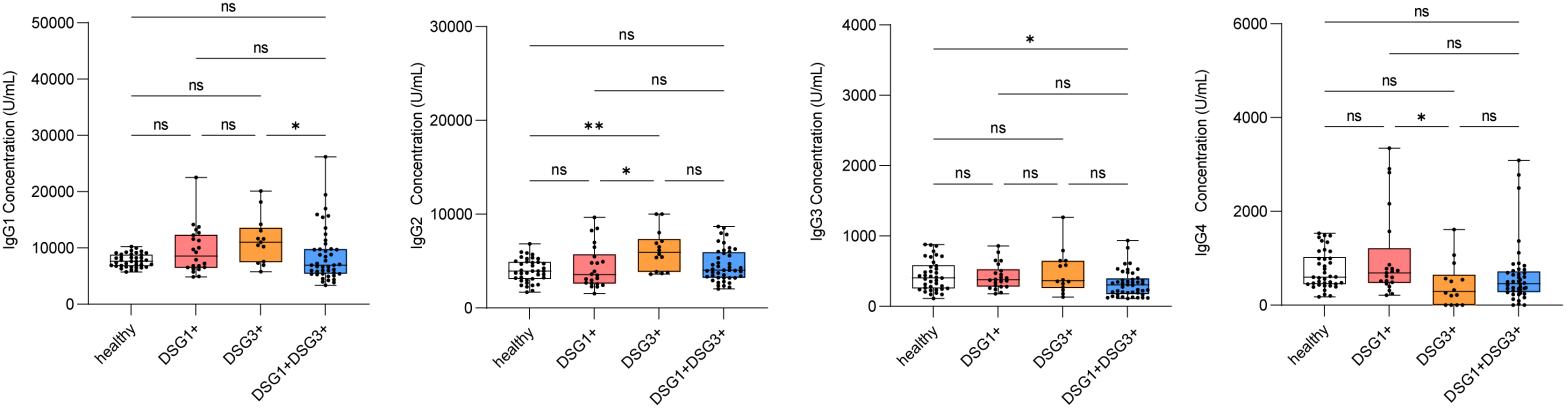
Differential analysis of IgG subclasses among pemphigus patients with different antibody-positive statuses during the active stage. Box plots combined with scatter plots were used to show the distribution differences of serum IgG1, IgG2, IgG3, and IgG4 among healthy controls, anti-Dsg1 single-positive, anti-Dsg3 single-positive, and anti-Dsg1/Dsg3 double-positive patients at the active stage. ns, not significant; *P < 0.05, **P < 0.01.

Results from the active stage demonstrated that the serum IgG2 concentrations were significantly and specifically elevated in anti-Dsg3 single-positive patient. Moreover, the IgG4 concentrations in anti-Dsg3 single-positive patients were significantly lower compared with those in anti-Dsg1 single-positive patients. Additionally, the expression level of IgG3 in anti-Dsg1/Dsg3 double-positive patients was also significantly lower than that in healthy controls. These findings indicated that IgG subclasses primarily exhibit a significant correlation with Dsg3 antibody levels during the active stage of pemphigus (**Figure 4**).

**Figure 5:**
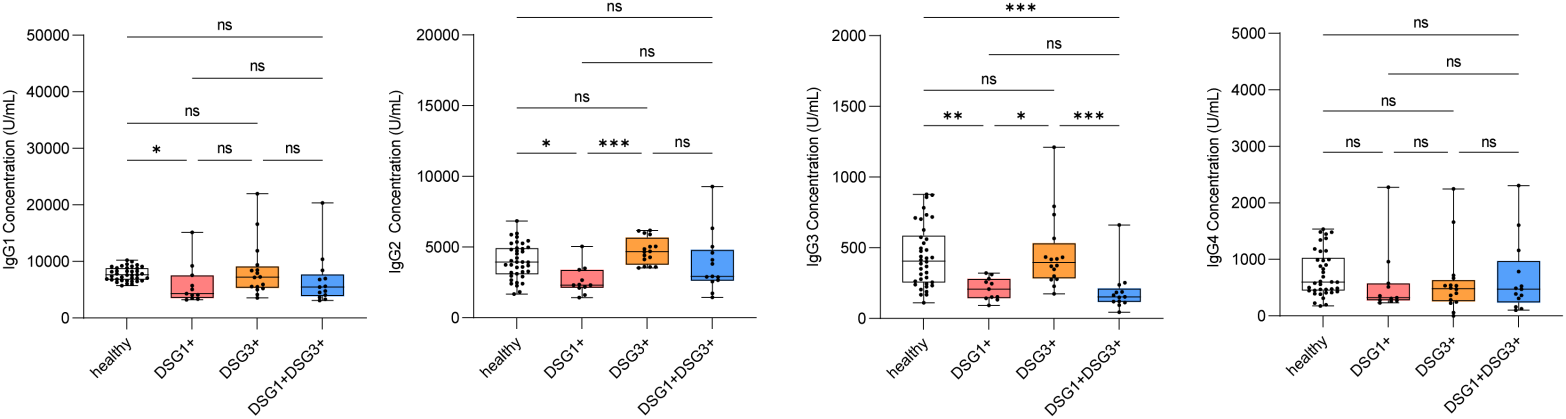
Differential analysis of IgG subclasses among pemphigus patients with different antibody-positive statuses during the remission stage. Box plots combined with scatter plots were used to show the distribution differences of serum IgG1, IgG2, IgG3, and IgG4 among healthy controls, anti-Dsg1 single-positive, anti-Dsg3 single-positive, and anti-Dsg1/Dsg3 double-positive patients at the remission stage. ns, not significant; *P < 0.05, **P < 0.01, ***P < 0.001.

In the remission stage of pemphigus, we found that the differences at this stage were mainly concentrated in anti-Dsg1 single-positive patients. Specifically, anti-Dsg1 single-positive patients showed a significant decrease in IgG1, IgG2, and IgG3, with a specific reduction in IgG2 and IgG3. Compared with anti-Dsg1 single-positive patients, the concentrations of IgG2 and IgG3 were significantly elevated in anti-Dsg3 single-positive patients. Anti-Dsg1/anti-Dsg3 double-positive patients were characterized by decreased IgG3 levels (**Figure 5**).

In summary, IgG subclasses demonstrate intergroup differences across different antibody-positive groups during various stages of the disease, thereby indicating their complex correlations with pemphigus.

### 3.3 Differential analysis of IgG1-IgG4 across different stages in patients with different antibody-positive subgroups

To gain a deeper understanding of the interplay between IgG subclasses and autoantibody levels across different disease statuses of pemphigus, we performed a comprehensive differential analysis of IgG1, IgG2, IgG3, and IgG4 levels across four distinct groups (healthy controls, baseline stage, active stage, and remission stage) within each antibody-positive subgroup.

**Figure 6:**
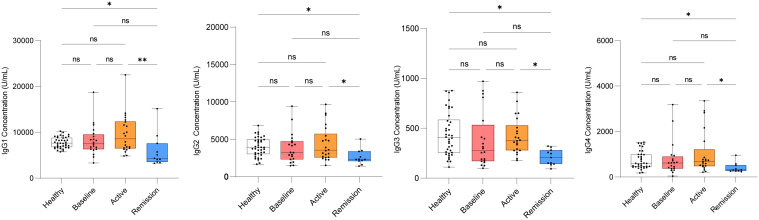
Differential analysis of IgG1-IgG4 in anti-Dsg1 single-positive patients across healthy controls, baseline, active, and remission stages. Box plots combined with scatter plots were used to show the differences in the distribution of serum concentrations of IgG1, IgG2, IgG3, and IgG4 among healthy controls, and anti-Dsg1 single-positive pemphigus patients at the baseline, active, and remission stages. ns, not significant; *P < 0.05, **P < 0.01.

In patients with anti-Dsg1 single-positivity, IgG1, IgG2, IgG3, and IgG4 concentrations were significantly lower in the remission group than in the healthy controls and active group (**Figure 6**). These findings suggest a strong correlation between all IgG subclasses and anti-Dsg1 antibodies, with lower levels characteristic of patients in remission.

**Figure 7:**
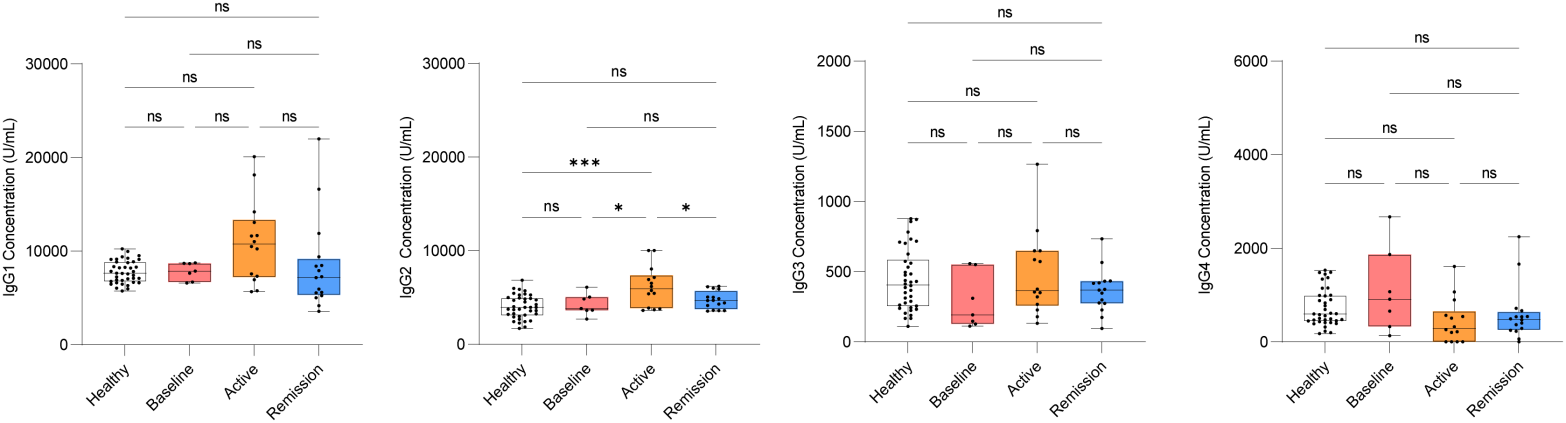
Differential analysis of IgG1-IgG4 in anti-Dsg3 single-positive patients across healthy controls, baseline, active, and remission stages. Box plots combined with scatter plots were used to show the differences in the distribution of serum concentrations of IgG1, IgG2, IgG3, and IgG4 among healthy controls, and anti-Dsg3 single-positive pemphigus patients at the baseline, active, and remission stages. ns, not significant; *P < 0.05, ***P < 0.001.

Conversely, in patients with anti-Dsg3 single-positivity, IgG2 levels were significantly higher in the active group than in the baseline group and healthy controls. IgG2 levels were markedly lower in the remission group compared with the active group (**Figure 7**). This distinct stage-dependent change pattern implies that IgG2 could serve as a valuable indicator for distinguish disease stages in this subgroup.

**Figure 8:**
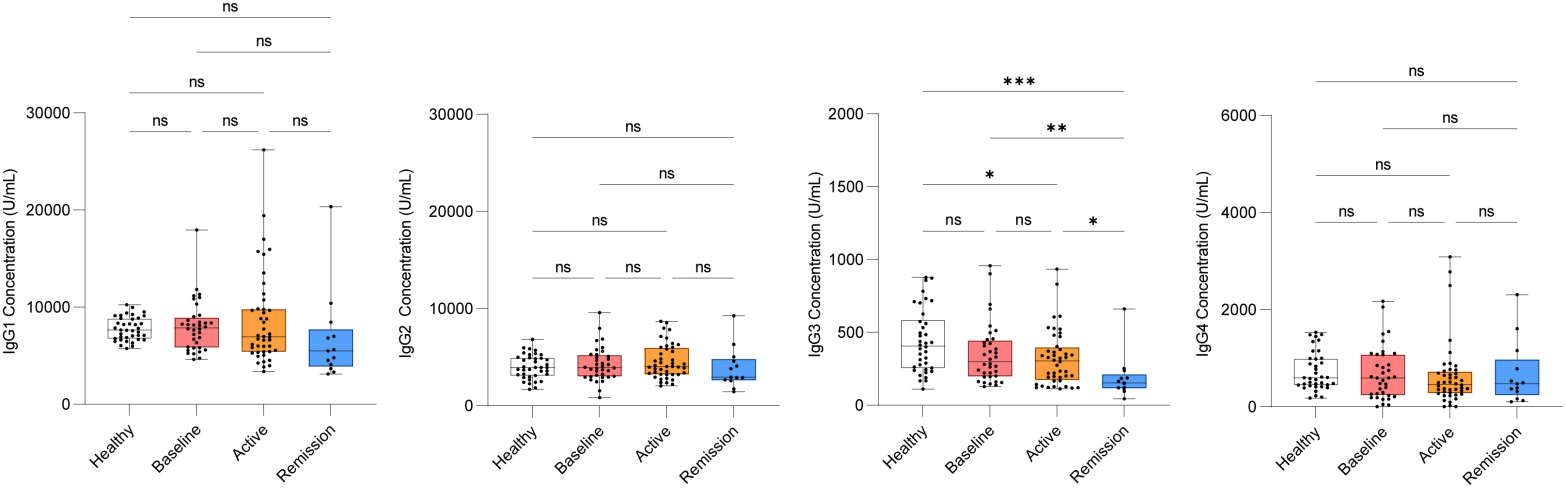
Differential analysis of IgG1-IgG4 in anti-Dsg1/Dsg3 double-positive patients across healthy controls, baseline, active, and remission stages. Box plots combined with scatter plots were used to show the differences in the distribution of serum concentrations of IgG1, IgG2, IgG3, and IgG4 among healthy controls, and anti-Dsg1/Dsg3 double-positive pemphigus patients at the baseline, active, and remission stages. ns, not significant; *P < 0.05, **P < 0.01, ***P < 0.001.

Moreover, in anti-Dsg1/Dsg3 double-positive patients, IgG3 levels were significantly lower in the active and remission groups compared to healthy controls, while no significant differences were noted in the other IgG subclasses (**Figure 8**). Importantly, this reduction in IgG3 levels may provide an additional indicator for assessing disease remission in patients positive for anti-Dsg1 antibodies.

**Figure 9:**
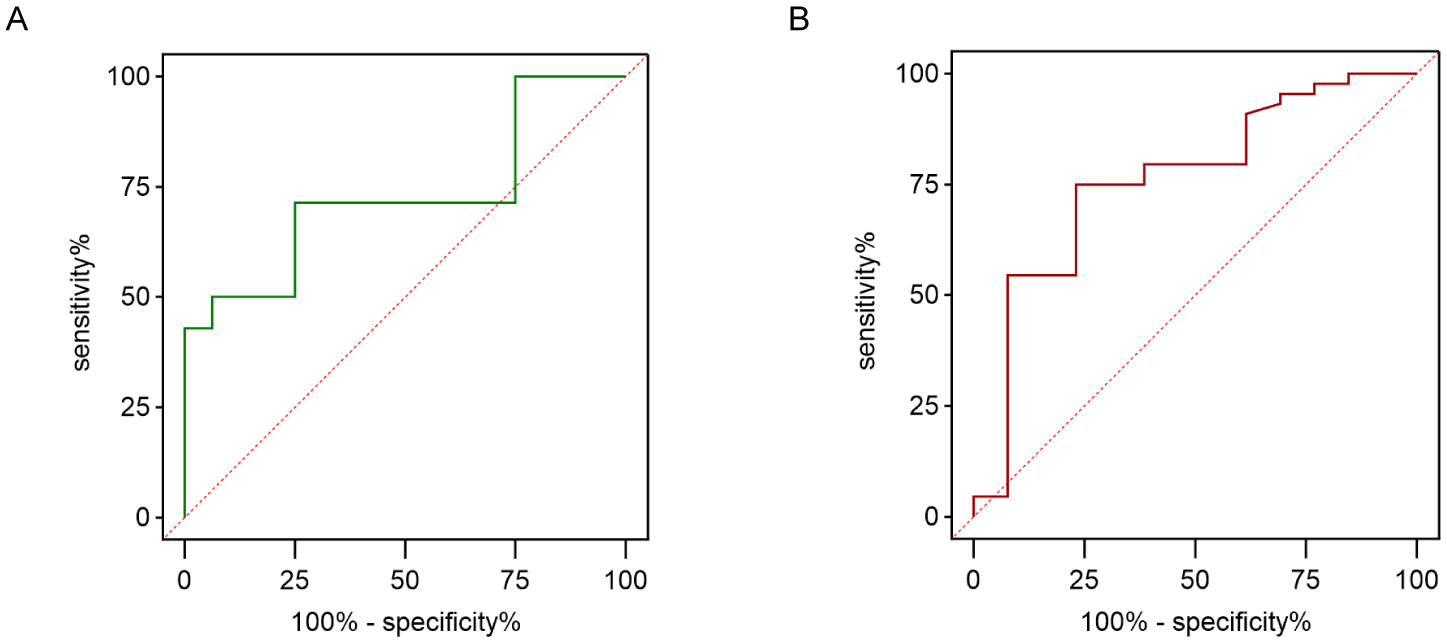
ROC curves of IgG2 and IgG3 for differentiating active and remission pemphigus. **(A)** ROC curve of IgG2 in patients with isolated anti-Dsg3 positivity. **(B)** ROC curve of IgG3 in anti-Dsg1/Dsg3 double-positive patients.

To further quantify the diagnostic performance of IgG2 and IgG3 for discriminating active and remission pemphigus, we performed receiver operating characteristic (ROC) curve analyses (**Figure 9**). Among them, in patients with isolated anti-Dsg3 positivity, IgG2 yielded an AUC of 0.7277 (95%*CI*: 0.5360-0.9194, *P* = 0.0340). The optimal cutoff value was 5224. IgG2 levels above this threshold indicated active pemphigus, with a sensitivity of 71.43% and specificity of 75.00% (Youden’s J index = 46.43) (**Table 2**). For anti-Dsg1/Dsg3 double-positive patients, IgG3 presented an AUC of 0.7596 (95%*CI*: 0.6039-0.9153, *P* = 0.0047). The optimal cutoff value was 189. IgG3 levels above this cutoff suggested active disease, with a sensitivity of 75.00% and specificity of 76.92% (Youden’s J index = 51.92) (**Table 2**). These cutoffs could be used to distinguish active and remission status based on IgG subclass concentrations.

**Table 2:** Diagnostic performance of IgG subclasses for distinguishing active and remission pemphigus in different autoantibody subgroups.

Study subgroup	Assay index	AUC	95% CI of AUC	P value	Optimal cutoff value	Sensitivity (%)	Specificity (%)	Youden’s J index
anti-Dsg3 positivity	IgG2	0.7277	0.5360-0.9194	0.0340	5224	71.43	75.00	46.43
anti-Dsg1/3 double positivity	IgG3	0.7596	0.6039-0.9153	0.0047	189	75.00	76.92	51.92

## 4 Discussion

Pemphigus is a chronic autoimmune bullous dermatosis mediated by autoantibodies. Its core etiology lies in the body’s production of IgG antibodies targeting Dsg1 and Dsg3 (1, 18). Previous studies indicated that anti-Dsg1 antibody levels can clearly reflect pemphigus disease activity (19). In contrast, anti-Dsg3 antibodies exhibit relatively low disease specificity, which imposes limitations on assessing disease status solely relying on anti-Dsg antibodies (15, 16). In present study, IgG subclasses levels were systematically detected and stratified analyzed across different disease stages and different pathogenic antibody-positive subgroups. Regional studies have characterized Ig and complement profiles in pemphigus, supporting the value of serological profiling in diverse populations (9). Consistent with *Elhag* et al., who observed altered immunoglobulin and complement levels in a Sudanese pemphigus cohort, our results indicate systemic humoral changes associated with disease activity (9). We found that IgG3 was specifically decreased during remission in anti-Dsg1-positive patients. Meanwhile, IgG2 levels were abnormally elevated during the active stage in anti-Dsg3 single-positive patients compared to other stages. These results provide precise serological tools for distinguishing disease stages and individualized clinical assessment, serving as an effective complement to the Dsg antibody.

IgG3 is the often first IgG subclass a B cell switches to from early primary and autoimmune response-derived IgM and the strongest effector functions among all four IgG subclasses (20). Previous studies have reported that the p.Arg435His variation in IgG3 could be associated with susceptibility to pemphigus, suggesting an unknown role for the function of IgG3 in the pathogenesis of pemphigus (21). Meanwhile, IgG3 is the strongest complement activation ability, and existing studies have confirmed that complement activation is directly associated with epidermal cell membrane damage in pemphigus vulgaris (PV) and pemphigus foliaceus (PF) (22–24). In anti-Dsg1 antibody-positive patients, inflammation resolves during the remission stage, the demand for complement activation decreases, and the levels of IgG3 decline accordingly. This suggests that in clinical practice, IgG3 concentrations can be detected to assess disease remission.

There have been few previous reports concerning IgG2 in pemphigus (25). In our study, IgG2 concentrations differed by disease stage among patients with isolated anti-Dsg3 positivity, with higher levels observed in active-stage patients and normal levels in remission-stage patients. This is the first time we have identified a potential association between IgG2 and Dsg3 autoantibodies, which provides a novel tool for discriminating disease stages. This fills the gap where anti-Dsg3 antibodies cannot accurately assess the disease status.

It has confirmed that Dsg-specific autoantibodies are highly enriched in the IgG4 subclass (26, 27). On the basis of the reactivity of IgG subclasses with various peptides spanning the extracellular domain of the PV antigen, *Bhol* et al. concluded that IgG4 is the main pathogenic antibody in PV (28). Meanwhile, PV patients who lack response to rituximab fail to reduce both IgG1 and IgG4 anti-Dsg1 antibodies further supports the pathogenicity of both subclasses (29). Consistent with this, our study further found that the levels of IgG1 and IgG4 were significantly decreased at the remission stage in anti-Dsg1 single-positive patients. This clearly reveals the potential association between IgG1, IgG4, and Dsg1-mediated disease activity.

This study still has certain limitations. First, this cross-sectional retrospective study adopted single-point sampling of distinct patients and could not track longitudinal changes in individual IgG subclasses. Second, adjustments were not made for confounders (age, disease duration, steroid dose and treatment length, concurrent medications, and therapeutic regimens), which to some extent weakens the reliability of our experimental conclusions. Finally, the predictive value of IgG subclasses for pemphigus relapse remains unvalidated. We intend to construct a long-term follow-up cohort in subsequent research, which will allow us to explore the correlations between IgG subclasses, disease progression and relapse, and screen cutoff values suitable for clinical application.

*Elhag* et al.’s findings reinforce that complement and immunoglobulin measurements could complement antigen-specific antibody assays when evaluating disease course (9). In present study, we further identified preliminary cutoff values of IgG2 for anti-Dsg3 single-positive patients and IgG3 for anti-Dsg1/Dsg3 double-positive patients to distinguish active and remission pemphigus. The two quantitative biomarkers identified in this study complement routine anti-desmoglein antibody assays. Combined detection of IgG2 and IgG3 with matched cutoff values during follow-up provides reference thresholds for disease stratification. Persistently high levels signal active disease and require slower steroid tapering or additional immunosuppressants. Sustained levels below the threshold indicate stable remission and permit gradual dose reduction to minimize overtreatment side effects (17). This stratified cut-off system provides quantitative laboratory evidence for individualized care of pemphigus with distinct antibody profiles.

In conclusion, IgG subclasses are the first to be analyzed across different disease stages and different pathogenic antibody-positive subgroups. IgG3 was specifically decreased during remission in anti-Dsg1-positive patients. Meanwhile, the levels of IgG2 exhibit a significant increase during the active stage in anti-Dsg3 single-positive patients. Measurement of the aforementioned IgG subclasses will provide effective serological tools for distinguishing disease stages and individualized clinical evaluation, serving as a valuable complement to existing Dsg antibody assays.

## Data Availability

The raw data supporting the conclusions of this article will be made available by the authors, without undue reservation.
